# Increased intestinal *Lactobacillus* abundance in post-pancreatectomy steatotic liver disease is associated with altered bile acid metabolism and FXR–FGF19 pathway suppression

**DOI:** 10.1080/29933935.2025.2607927

**Published:** 2025-12-27

**Authors:** Kohta Iguchi, Natsumi Seki, Yuki Sugiura, Rae Maeda, Kenzo Nakano, Takayuki Kawai, Yukihiro Okuda, Ryo Kamimura, Yoichiro Uchida, Akihiro Hamasaki, Kojiro Taura, Hiroaki Terajima

**Affiliations:** a Department of Gastroenterological Surgery and Oncology, Medical Research Institute KITANO HOSPITAL, PIIF Tazuke-Kofukai, Osaka, Japan; b Center for Cancer Immunotherapy and Immunobiology, Graduate School of Medicine, Kyoto University, Kyoto, Japan; c Human Biology Microbiome Quantum Research Center (WPI-Bio2Q), Keio University School of Medicine, Tokyo, Japan; d Department of Surgery, Kyoto City Hospital, Kyoto, Japan; e Department of Surgery, Toyooka Hospital, Toyooka, Hyogo, Japan; f Department of Surgery, Graduate School of Medicine, Kyoto University, Kyoto, Japan; g Department of Diabetes and Endocrinology, Tazuke Kofukai Medical Research Institute, Kitano Hospital, Osaka, Japan

**Keywords:** Post-pancreatectomy steatotic liver disease, bile acid, bile salt hydrolase, fibroblast growth factor 19, gut microbiota, metabolomics, nonalcoholic fatty liver disease, metabolic dysfunction-associated steatotic liver disease

## Abstract

Steatotic liver disease, a common metabolic disorder characterized by hepatic fat accumulation, is frequently associated with altered bile acid metabolism. Post-pancreatectomy steatotic liver disease (PPSLD) develops in approximately 37% of patients undergoing pancreaticoduodenectomy and can progress to steatohepatitis, liver failure, or death. Here, we investigated microbial, bile acid, and metabolic features associated with PPSLD across two clinical cohorts. Patients with PPSLD exhibited a consistent increase in facultative anaerobes, particularly *Lactobacillus* species, together with an increased predicted capacity for bile acid deconjugation. This microbial profile was associated with higher circulating levels of deconjugated bile acids and a biochemical pattern suggestive of attenuated intestinal FXR–FGF19 signaling and increased hepatic bile acid synthesis. In addition, patients with PPSLD exhibited lower plasma choline and carnitine levels, indicating reduced availability of nutrients essential for phospholipid and fatty acid metabolism. These findings suggest that microbial dysbiosis, bile acid dysregulation, and postoperative nutrient limitations are associated with the PPSLD phenotype, in addition to pancreatic exocrine insufficiency. This study identifies integrated microbial and metabolic alterations associated with PPSLD. While causal relationships cannot be inferred from these observational data, our results may inform future investigations into therapeutic strategies aimed at maintaining metabolic and microbial homeostasis in post-pancreatectomy patients.

## Graphical abstract

**Figure:**
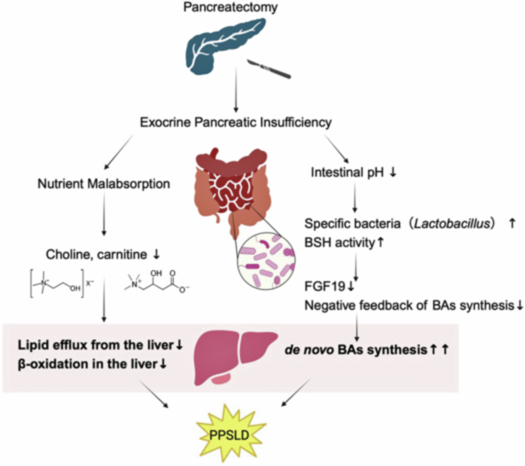

## Introduction

Pancreatic resection is the cornerstone treatment for pancreatic cancer and can significantly improve patient survival.^1^ However, this procedure is highly invasive and frequently results in complications, such as glucose intolerance, exocrine pancreatic insufficiency, and malnutrition caused by impaired digestion and nutrient absorption. One notable postoperative complication is post-pancreatectomy steatotic liver disease (PPSLD), which is reported in approximately 37% of patients who undergo pancreaticoduodenectomy (PD).^2^ Onset can be detected using computed tomography (CT) as early as 1–3 months postoperatively, and pancreatic enzyme replacement therapy (PERT) is typically administered.^3^ Although most patients respond to PERT within one year, refractory cases may progress to steatohepatitis, liver failure, or even death.^4^

Intriguingly, PPSLD has been observed predominantly after PD or total pancreatectomy (TP)—both of which involve gastrointestinal and biliary reconstruction—whereas distal pancreatectomy (DP), which does not require such reconstruction, generally leads to PPSLD only rarely.^2^^,^^5^ Even in DP, PPSLD is uncommon unless the remnant pancreas is extremely small, suggesting that both the extent of pancreatic resection and surgery-induced changes in the gastrointestinal environment may contribute to PPSLD development, although their relative contributions remain unclear.

Recent findings indicate that the gut microbiota plays a pivotal role in metabolic dysfunction-associated steatotic liver disease (MASLD),^6–8^ partly by modulating bile acid metabolism. Alterations in bile acid composition can affect the activation of the farnesoid X receptor (FXR),^9^ which in turn regulates intestinal lipid absorption and hepatic lipogenesis through fibroblast growth factor (FGF)19-mediated negative feedback.^9–11^

After pancreatic resection, the gut microbiota is expected to shift substantially due to antibiotic exposure, altered gastrointestinal pH, and exocrine insufficiency. However, the mechanism by which these post-resection microbial changes contribute to PPSLD has not yet been systematically explored.

In this study, we hypothesized that gut dysbiosis in the post-pancreatectomy setting is associated with disturbed bile acid homeostasis and impaired FXR–FGF19 signaling, which in turn are linked to altered hepatic lipid metabolism in PPSLD. To explore this hypothesis, we performed comprehensive analyzes encompassing gut microbiota profiles in fecal samples, bile acid measurements in blood samples collected at multiple time points, and bile acid imaging of liver tissue obtained at a specific time point between resection and PPSLD onset. Our results suggest that characteristic microbial alterations and changes in bile acid homeostasis are associated with the PPSLD phenotype.

## Materials and methods

### Patients

**Figure 1.**
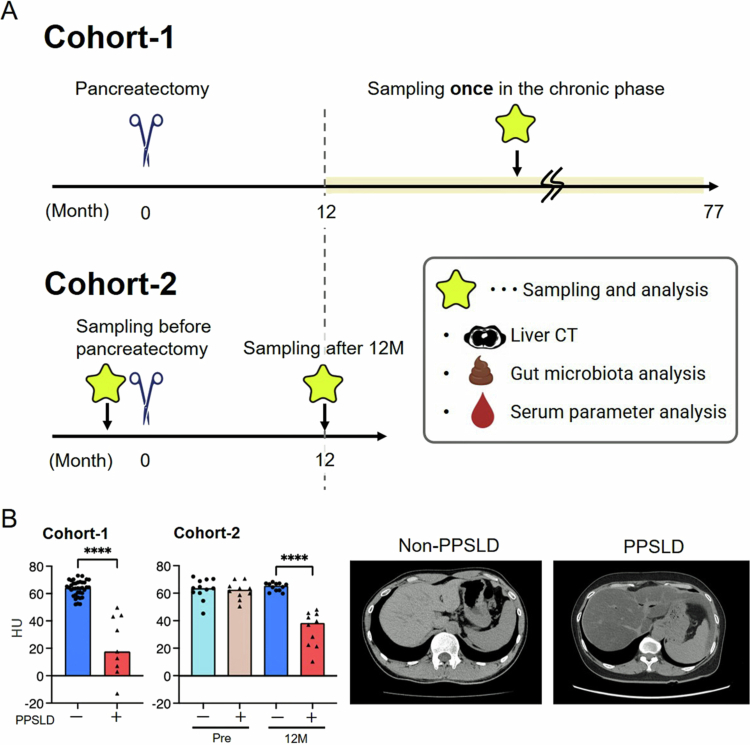
Study design. (A) Study design. (B) A comparison of liver CT values between groups on unenhanced CT and representative unenhanced CT images. *Cohort-1 was a retrospective study that enrolled patients who had previously undergone pancreatic resection. This was a chronic-phase cohort with a wide range of postoperative time (12–77 months, median 31 months). *Cohort-2 is a prospective study that enrolled patients scheduled to undergo PD or TP associated with a higher risk of PPSLD. Sampling was performed at two time points: preoperatively (pre) and 12 months postoperatively (12 M), making this an acute-phase cohort. *Patients in Cohort-2 received comprehensive dietary and nutritional counseling based on detailed dietary surveys conducted by a registered dietitian from the preoperative period through one year postoperatively. *PPSLD was defined as a liver CT value of 50 Hounsfield units or less on plain CT. The darker the liver density on CT, the more severe the degree of fatty liver. CT, computed tomography; PD, pancreaticoduodenectomy; PPSLD, post-pancreatectomy steatotic liver disease; TP, total pancreatectomy.

Two cohort studies were conducted (Figure 1A). A cohort of patients who had previously undergone pancreatic resection was selected to identify the characteristics of patients with refractory PPSLD (Cohort-1). This cohort included patients in the chronic phase with a median postoperative follow-up period of 31 months (12–77 months). Furthermore, to verify the validity of the obtained results, we conducted a study (Cohort-2) that observed changes from preoperative to 12 months postoperatively in the same patients. In Cohort-2, intensive nutritional counseling interventions were implemented to prevent PPSLD.

1. Cohort-1

In total, 180 patients were identified who underwent pancreatectomy at the Medical Research Institute, Kitano Hospital, between January 2014 and February 2020 (Figure S1). As of June 2020, 141 patients were alive, and 117 actively attended our outpatient clinic. Among these, nine patients with pre-existing liver disease, including MASLD or kidney disease requiring dialysis, and 13 patients undergoing chemotherapy for their primary disease were excluded. Consequently, 95 patients were included in the final analysis. Finally, 47 patients who visited our outpatient clinic between June and December 2020 and provided informed consent were enrolled.

2. Cohort-2

From July 2020 to September 2021, 25 patients scheduled for PD or TP at Kitano Hospital who provided informed consent were prospectively enrolled and followed up for one year (Figure S1). Patients with pre-existing liver or kidney disease were excluded from the study.

This study was approved by the Ethics Committee of Kitano Hospital (P200600802).

### Surgery

In patients with PD, a subtotal stomach-preserving approach (subtotal stomach-preserving PD) was employed. The pancreatic resection line was determined intraoperatively using ultrasonography to localize the tumor, and the absence of malignancy was verified through intraoperative consultation. Dissection of the first jejunal vein and resection of the first jejunal artery have been consistently performed in cases of pancreatic cancer. The extent of jejunal resection varied according to the vascular territory supplied by the first jejunal artery and first jejunal vein, with an average resection length of approximately 10 cm.

Surgical reconstruction following PD was performed using Child’s method, which included pancreaticojejunostomy, choledocojejunostomy, and gastrojejunostomy with Braun anastomosis. Pancreaticojejunostomy was performed using a modified Blumgart technique, with duct-to-mucosa anastomosis achieved through hand-sewn interrupted sutures with 6-0 monofilament absorbable sutures, and a 4 Fr pancreatic duct stent was placed as a lost stent. Choledocojejunostomy was performed with hand-sewn interrupted sutures using 5-0 monofilament absorbable sutures without using stents.

### Postoperative management

In the immediate postoperative period, patients received intravenous amino acid infusions until oral intake was deemed feasible. Oral intake began with a liquid diet on postoperative day four and progressed to a porridge diet on postoperative day five. After hospital discharge, amino acid supplementation with Elental® (EA Pharma, Japan) was typically continued for six months. Notably, none of the patients in Cohort-1 received Elental during the study period. All patients were prescribed oral proton pump inhibitors.

Patients who underwent PD or TP received oral zinc supplementation, whereas those who underwent DP did not. The standard dose of pancrelipase delayed-release capsules (LipaCreon®, Mylan Inc., PA, U.S.A) was 1.8 g/day for patients undergoing PD or TP and 1.35 g/day for those undergoing DP. Dose adjustments were made based on individual nutritional status and laboratory test results.

### Evaluation of PPSLD on CT images

CT was performed to assess the presence or absence of steatosis in the liver. PPSLD was defined as a liver CT attenuation value of 50 Hounsfield units (HU) or less on non-contrast CT scans^12^^,^^13^ (Figure 1B). Four regions of interest (ROIs) were selected to avoid the major vessels, with one ROI each placed in the lateral, medial, anterior, and posterior segments of the liver. Average CT attenuation values were calculated from the four ROIs. Each ROI had a surface area of approximately 1.0 cm².

### Sample collection

1. Cohort-1

Fasting blood and stool samples were collected after obtaining informed consent (Figure 1A). Patients who were taking LipaCreon®, ursodeoxycholic acid (UDCA), or probiotics discontinued these medications four days before sample collection to minimize potential confounding effects on the variables being evaluated. In cases where antibiotics were administered for fever due to cholangitis, sample collection was delayed for at least one month to account for potential effects on the fecal microbiome. Blood samples were centrifuged, and the resulting serum was stored at –80 °C for future analysis. Fecal samples were collected using a fecal sampling kit (TechnoSuruga Laboratory Co. Ltd., Shizuoka, Japan).

2. Cohort-2

Fasting blood and stool samples were collected before surgery (Pre) and 12 months (12 M) postoperatively (Figure 1A). Three patients who developed obstructive jaundice before surgery and underwent endoscopic nasobiliary drainage, one patient with ulcerative colitis, two patients with a history of bowel resection, and three patients who died within 12 months of surgery were excluded from the preoperative fecal analysis.

### Clinical characteristics and serum parameters

In Cohort-1, patients were divided into “PPSLD” and “non-PPSLD” groups based on the presence or absence of PPSLD. In Cohort-2, the same parameters as in Cohort-1 were compared between the “Pre” and “12M” groups. In both cohorts, the percentage of residual pancreatic volume was calculated using image analysis software (Volume Analyzer SYNAPSE VINCENT; Fujifilm Medical Co. Ltd., Tokyo, Japan), which determined the ratio of pancreatic volumes measured on CT before surgery and at the time of this study.^14^ Steatorrhea was evaluated using Sudan III staining. In addition, the dosage of LipaCreon® and the use of antihyperlipidemic agents or insulin were evaluated. Blood biochemistry tests were performed to assess various lipid and energy metabolism indices and protein synthesis indices. Liver function markers and the fibrosis-4 (FIB-4) index, an indicator of liver fibrosis, were also calculated.^15^ Zinc levels, critical for maintaining exocrine pancreatic function, were measured.^16^

At 12 M, patients were further divided into “12M PPSLD” and “12M non-PPSLD” groups based on the presence or absence of PPSLD at that time. The preoperative status of these groups was referred to as the “Pre-PPSLD” group and the “Pre-non-PPSLD” group, respectively.

### Body composition analysis

Skeletal muscle was measured on axial CT images at the level of the umbilicus using HU thresholds ranging from –19 to + 150. The iliopsoas muscle was manually traced. ROIs were analyzed using SYNAPSE VINCENT software, and muscle mass was normalized by dividing it by the square of the patient’s height (m²). In addition, bioelectrical impedance analysis (InBody S10, InBody, Japan) was used to assess body fat percentage.

### Nutritional assessment

In Cohort-2, patients received nutritional counseling from dietitians at seven time points: preoperatively, before discharge, and 1.5, 3, 6, 9, and 12 months postoperatively. In Cohort-1, only patients with PPSLD were interviewed to assess their current dietary habits. Dietitians conducted interviews and analyzed the dietary records using Excel Eiyo-kun version 9.0 and FFQg (version 6.0) (Kenpaku Co. Ltd., Tokyo, Japan). They estimated all ingredients and their weights and calculated protein, fat, and carbohydrate intake. The FFQg, widely used in epidemiological cohort studies, provides the average weekly intake data for 29 food groups and 10 cooking methods in conventional units or portion sizes.^17^ The final nutrient intake was determined by averaging the values from both the food records and FFQg to address the limitations of each method.

### Analysis of fecal microbiome

DNA was extracted as described previously.^18^ An automated DNA isolation system (GENE PREP STAR PI-480; KURABO, Tokyo, Japan) was used for this process. The V3-V4 bacterial and archaeal 16S rRNA gene regions were amplified using Pro341F/Pro805R primers and the dual-index method.^18^^,^^19^ Barcoded amplicons were sequenced by paired-end sequencing with a 2 × 301-bp cycle on a MiSeq system using the MiSeq Reagent Kit version 3 (600 cycle chemistry). Sequencing data were processed and analyzed using Qiime2 (version 2024.5).^20^ Primers were trimmed using the Cutadapt plugin in Qiime2 (https://doi.org/10.14806/ej.17.1.200). After primer removal, the resulting sequences were subjected to quality control, paired-end joining, chimera filtering, and ASV table construction using the DADA2 algorithm.^21^ Taxonomy was assigned to each representative ASV sequence using BLAST^22^ based on the SILVA database (version 138).^23^ A random sampling of 10,000 reads was performed using a feature table,^24^ followed by compositional data conversion and diversity analysis. The functional potential of the gut microbiota was predicted using PICRUSt2 (https://www.nature.com/articles/s41587-020-0548-6). PICRUSt2 analysis was performed using Qiime2 version 2023.2 due to the incompatibility of PICRUSt2 with Qiime2 version 2024.5.

### Measurement of bile acids

Bile acid concentrations in serum samples were quantified using liquid chromatography coupled with mass spectrometry (LC-MS). The 50 µL of serum was mixed with 500 μL of methanol containing internal standards: cholic acid-d4 (d4-CA), chenodeoxycholic acid-d4 (d4-CDCA), taurocholic acid-d5 (d5-TCA), taurochenodeoxycholic acid-d4 (d4-TCDCA), glycocholic acid-d4 (d4-GCA), deoxycholic acid-d4 (d4-DCA), lithocholic acid-d4 (d4-LCA) (all from Cayman Chemical Company, MI, U.S.A), and glycodeoxycholic acid-d4 (d4-GDCA) (Santa Cruz Biotechnology Inc., CA, U.S.A). Then, the samples were centrifuged at 16,000 × *g* for 5 min at 4 °C. The resulting supernatant was collected, diluted with methanol, and subjected to LC-MS analysis.

The LC-MS system used was an LCMS-8060 (Shimadzu Co., Kyoto, Japan) equipped with the LC/MS/MS Method Package for Bile Acids (Shimadzu Co.), which provided information on the LC analytical conditions and multiple reaction monitoring parameters for bile acid analysis. In this study, the default LC conditions from the package were modified as follows: Mobile phase B was adjusted to acetonitrile/methanol (50:50). Data processing, including peak alignment and identification, was performed using LabSolutions Insight for LC-MS (Shimadzu Co.). Bile acid concentrations were calculated using the internal standard method, based on the peak area ratios of bile acids to their corresponding internal standards.

### Liquid chromatography-tandem mass spectrometry for metabolite measurement

Following established protocols, serum metabolite concentrations were quantified using liquid chromatography-tandem mass spectrometry (LC-MS/MS).^25^ The analysis used a triple-quadrupole mass spectrometer (LCMS-8060, Shimadzu Co.) equipped with an electrospray ionization (ESI) source. The instrument was operated in both positive and negative ESI modes using multiple reaction monitoring (MRM).

Analyte separation was conducted on a Discovery HS F5-3 column (2.1 mm I.D. × 150 mm, 3 μm particle size; Sigma-Aldrich, St. Louis, MO, U.S.A) using gradient elution. The mobile phase consisted of solvents A (0.1% formate in water) and B (acetonitrile with 0.1% formate). The gradient profile was as follows: 100% A (0–5 min), 75% A/25% B (5–11 min), 65% A/35% B (11–15 min), 5% A/95% B (15–20 min), and 100% A (20–25 min). The flow rate was maintained at 0.25 mL/min, and the column oven temperature was set to 40 °C.

### Serum FGF19 levels in Cohort-1

Serum FGF19 levels were determined using a commercially available human FGF19 sandwich ELIZA kit (ab230943; Abcam, Cambridge, UK) according to the manufacturer’s instructions.

### Immunohistochemical staining of hepatic CYP7A1 in a PPSLD case from Cohort-1

Liver tissue was obtained from a single patient with PPSLD who underwent liver resection for a recurrent portal vein tumor thrombus after PD for a duodenal gastrointestinal stromal tumor. Control liver tissue was obtained from one non-PPSLD patient with colorectal cancer liver metastasis, whose background liver was pathologically normal and who had received no preoperative treatment. For CYP7A1 immunohistochemistry, liver tissues were formalin-fixed, paraffin-embedded, and sectioned at 4 μm. After deparaffinization and rehydration, antigen retrieval was performed by heating the slides in EDTA solution (Nichirei Biosciences, Tokyo, Japan; code 415211, pH 9) at 98 °C for 20 min. Endogenous peroxidase activity was blocked by treating the sections with 3% hydrogen peroxide for 10 min. After a 60-minute blocking step with 3% bovine serum albumin in phosphate-buffered saline, the sections were incubated overnight at 4 °C with a primary antibody against human CYP7A1 (18054-1-AP; Proteintech Group) at a 1:150 dilution. After washing with TBST, the sections were incubated with a peroxidase-conjugated anti-rabbit IgG antibody (Histofine Simple Stain MAX PO(R), code 724142; Nichirei Biosciences) for 30 min at room temperature. Staining was developed with a DAB substrate kit (code 725191; Nichirei Biosciences) and counterstained with hematoxylin.

### DESI-based imaging mass spectrometry

DESI-MRM data were acquired by integrating a Waters DESI-XS ionization source with a Waters Xevo TQ Absolute (TqA) mass spectrometer. Analysis was performed on one representative liver tissue sample from a patient with PPSLD and one from a non-PPSLD control. Methanol/water (98:2 v/v) was delivered from the Waters nanoAcquity Binary Solvent Manager through an M-Class Symmetry C18 column to the DESI sprayer at a 2 μL/min flow rate. A high voltage applied to the sprayer generated an electrospray that was pneumatically directed at a 75 ° angle toward the tissue section using a nitrogen gas stream, and the MRM parameters (*m/z* 307.76 > 178.81) were set in positive ion detection mode. The DESI spray voltage, heater transfer line temperature, and nitrogen gas flow were set to 0.8 kV, 150 °C, and 0.15 MPa, respectively, to ensure optimal performance in negative ionization mode. The optimized settings were saved in a Waters software. ipr file. Before the biological sample analysis, the motion and precise alignment of the x- and y-stages in the optimized DESI-MRM setup were verified using black ink (*m/z* 666.06) as the test analyte. The DESI-MRM experimental configuration was completed using HDImaging v1.7 and the DESI Method Editor (Waters).

### Statistics

Patient characteristics were compared between the “PPSLD” and “Non-PPSLD” groups in Cohort-1 and between the “Pre” and “12M” and the “12M PPSLD” and “12M non-PPSLD” groups in Cohort-2. Categorical variables were analyzed using the chi-squared test. Continuous variables are reported as median (range) and compared using either Student’s *t*-test or the Mann–Whitney *U* test, depending on the data distribution. Changes in continuous variables over time (Pre vs. 12 M) in Cohort-2 were evaluated using the Wilcoxon signed-rank test.

To assess determinants of PPSLD, a multivariable logistic regression analysis was performed within the PD subset of Cohort-1. Associations between remnant pancreas proportion and liver attenuation, bile acids, *Lactobacillus*, and FGF19 were examined using Spearman’s rank correlation. As an additional subgroup comparison, chemotherapy versus non-chemotherapy differences in bile acids and *Lactobacillus* in PD cases were evaluated using the Mann–Whitney *U* test. All *p*-values were two-tailed, and statistical significance was set at *p* < 0.05. Statistical analyzes were performed using IBM SPSS Statistics version 29.0.0.

## Results

### Clinical characteristics of PPSLD across two cohorts and associations with remnant pancreatic volume

In Cohort-1, which included patients with relatively stable long-term postoperative courses (median 31 months after surgery), 9 of 47 patients (19.1%) were classified as having PPSLD, whereas 38 (80.9%) did not show PPSLD (Table 1). Most PPSLD cases occurred in patients with invasive pancreatic ductal adenocarcinoma (PDAC; 66.7%) who underwent either PD (77.8%) or TP (22.2%). In contrast, no cases of PPSLD were observed after DP, which does not require duodenal resection. The PPSLD group had a significantly smaller residual pancreatic volume (22.8% vs. 40.5%) and more frequently received postoperative adjuvant chemotherapy (77.8% vs. 31.6%) than the non-PPSLD group.

**Table 1.** Patients’ characteristics in Cohort-1.

Clinical data		Non-PPSLD	PPSLD	*p*-value
		(*n* = 38)	(*n* = 9)	
Age (years)		74 [32–89]	72 [63–81]	0.76
Sex (male)		17 (44.7%)	4 (44.4%)	0.98
Disease (PDAC)		10 (26.3%)	6 (66.7%)	**0.03**
Preoperative chemotherapy (+)		0 (0%)	2 (22.2%)	0.3
Surgical procedure	PD	22 (57.9%)	7 (77.8%)	0.001*
	TP	0 (0%)	2 (22.2%)	
	DP	16 (42.1%)	0 (0%)	
Remnant volume of the pancreas (%)		40.5 [20.2–93.1]	22.8 [0–36.5]	0.0008*
Postoperative period (months)		31 [11–77]	31 [14–73]	0.57
Postoperative chemotherapy (+)		9 (23.7%)	7 (77.8%)	0.003*
Dose of pancrelipase capsules (g/day)		1.35 [0–2.25]	1.80 [1.35–2.70]	0.01*
Exogenous insulin use (+)		3 (7.9%)	2 (22.2%)	0.24
Use of antihyperlipidemic agents (+)		8 (21.1%)	1 (11.1%)	0.67
Steatorrhea (+)		1/37 (2.7%)	2/7 (28.6%)	0.03*
Use of antidiarrheal agents (+)		1 (2.6%)	0 (0%)	0.51

*The PPSLD group consists of six patients with PDAC, one with distal bile duct cancer, one with duodenal GIST, and one with IPMN. *The non-PPSLD group includes 10 patients with PDAC, eight with ampullary carcinoma, seven with pancreatic neuroendocrine tumors, six with mucinous cystic neoplasms, six with IPMN, and one with serous cystic neoplasm. DP, distal pancreatectomy; GIST, gastrointestinal stromal tumor; IPMN, intraductal papillary mucinous neoplasm; PD, pancreaticoduodenectomy; PDAC, pancreatic ductal adenocarcinoma; PPSLD, post-pancreatectomy steatotic liver disease; TP, total pancreatectomy.

Despite a higher daily intake of pancrelipase capsules in the PPSLD group (1.80 vs. 1.35 g/day), some patients remained refractory even at the maximum dose (2.70 g/day). Because no PPSLD events occurred after DP, subsequent determinant analyzes were restricted to patients who underwent PD.

In Cohort-2, evaluated at 12 months after surgery, PPSLD was diagnosed in 45.5% of patients. Although intensive nutritional counseling improved serum protein levels at 12 months, total cholesterol and triglyceride levels significantly decreased, and patients exhibited reduced muscle mass and lower body fat percentage, indicating a malnourished phenotype (Table S1). The median age was 71 years, and 44% of the participants were male, comparable to Cohort-1 (Table 2). PDAC was the most common pathology (60%), with 36% of the patients receiving neoadjuvant chemotherapy (gemcitabine + TS-1) and 36% receiving adjuvant chemotherapy (TS-1). Surgical procedures included PD in 20 patients and TP in five patients.

**Table 2.** Patients’ characteristics in Cohort-2.

All participants in Cohort-2		*n* = 25
Age (years)		71 [47–86]
Sex (male)		11 (44.0%)
Disease (PDAC)		15 (60.0%)
Preoperative chemotherapy ( + )		9 (36.0%)
Surgical procedure	PD	20 (80.0%)
	TP	5 (20.0%)
Diameter of the main pancreatic duct (mm)		4.0 [2.0–13.0]
Pancreatic stiffness (hard pancreas)		8 (32.0%)
Remnant volume of the pancreas (%)		28.2 [0–66.8]
Duration of surgery (min)		466 [300–682]
Blood loss (mL)		124 [18–34]
Postoperative stay (days)		22 [13–34]
Pancreatic fistula ( ≥ grade B)		2 (8%)
Postoperative chemotherapy ( + )		9 (36.0%)
**Participants after 12 M**		***n* = 22**
PPSLD ( + )		10 (45.5%)
Dose of pancrelipase capsules (g/day)		1.35 [0.45–2.70]
Exogenous insulin use ( + )		6 (27.3%)
Use of antihyperlipidemic agents ( + )		0 (0%)
Steatorrhea ( + )		4 (18.2%)

*In addition to 15 patients with invasive PDAC, surgery was performed in two patients with duodenal GIST, five patients with IPMN, two patients with ampullary carcinoma, and one patient with pancreatic NET. *The diameter of the main pancreatic duct was measured intraoperatively at the margin of the pancreatic transection (usually just above the portal vein). Patients who underwent TP were excluded. *Pancreatic stiffness is a subjective finding based on palpation by the surgeon; this information was extracted from operative records. *Pancreatic fistulas were classified according to the ISGPS definition and grading system.^26^ *Data at 12 months are missing for one patient who died 72 days after surgery due to acute exacerbation of postoperative interstitial pneumonia and two patients who died of other illnesses during the first year after surgery. GIST, gastrointestinal stromal tumor; IPMN, intraductal papillary mucinous neoplasm; ISGPS, International Study Group; NET, neuroendocrine tumor; PD, pancreaticoduodenectomy; PDAC, pancreatic ductal adenocarcinoma; PPSLD, post-pancreatectomy steatotic liver disease; TP, total pancreatectomy.

The median residual pancreatic volume was 28.2%. Among the patients with PPSLD at 12 months, 60% had received adjuvant chemotherapy, compared with 25% in the non-PPSLD group. Although this difference raised concerns regarding chemotherapy-related effects, the chronic nature of PPSLD in Cohort-1—where patients were sampled years after completion of adjuvant therapy—suggests that chemotherapy alone is unlikely to account for the observed phenotype.

A comparison of PPSLD severity between cohorts showed that Cohort-1 exhibited more pronounced hepatic steatosis. Mean unenhanced liver CT attenuation values were 64.2 HU (non-PPSLD) versus 17.7 HU (PPSLD) in Cohort-1, compared with 65.5 HU versus 38.4 HU at 12 months in Cohort-2 (Figure 1B). Paired trajectories from baseline to 12 months (Figure S2) demonstrated that postoperative declines in CT attenuation—rather than baseline variability—were characteristic of PPSLD.

To explore clinical factors associated with PPSLD independent of potential confounders, a multivariable logistic regression analysis was performed in the PD subset of Cohort-1 (Table S2). A higher remnant pancreas proportion (per 5-percentage-point increase) was associated with lower odds of PPSLD (adjusted OR 0.54, 95%CI 0.30–0.98, *p* = 0.044). Age, sex, BMI, and postoperative chemotherapy were not significant correlates in this model. These findings suggest that reduced remnant pancreatic volume is a strong clinical correlate of PPSLD within this cohort.

### Early elevation of serum bile acid levels and indicators of hepatic injury in PPSLD

**Figure 2.**
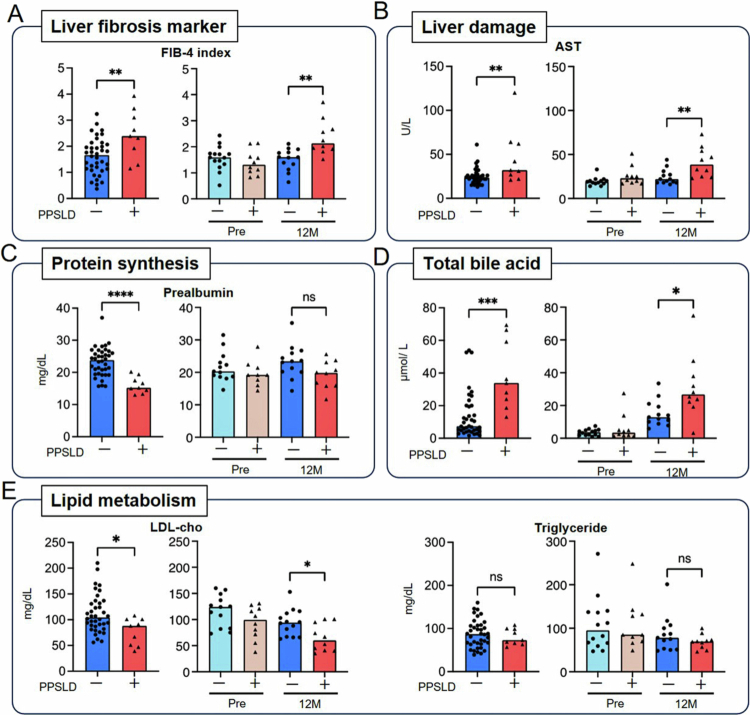
Representative variables associated with PPSLD. (A) FIB-4 index and levels of (B) AST, (C) prealbumin, (D) total bile acid, and (E) LDL-cho and triglyceride. *The FIB-4 index was calculated using the following formula: age × AST/platelet count [ × 10^3^/μL] × (ALT)^1/2^.^15^ *For each variable, the graph on the left represents Cohort-1, while the graph on the right represents Cohort-2. *Each dot represents one patient, and the bars indicate median values. Statistical significance was assessed using the Mann–Whitney *U* test or Student’s *t*-test. ^∗^*p* < 0.05; ^∗∗^*p* < 0.01; ^∗∗∗^*p* < 0.001; ^∗∗∗∗^*p* < 0.0001; ns, not significant. ALT, alanine aminotransferase; AST, aspartate aminotransferase; FIB-4, fibrosis-4; LDL-cho, low-density lipoprotein cholesterol; PPSLD, post-pancreatectomy steatotic liver disease.

Across both cohorts, the PPSLD group exhibited higher FIB-4 index values (Cohort-1: 2.39 vs. 1.67, *p* = 0.004; Cohort-2: 2.13 vs. 1.60, *p* = 0.002; Figure 2A), which are suggestive of potential liver fibrotic changes, although this marker is nonspecific and may also reflect hepatic injury. Serum aspartate aminotransferase (AST) levels were significantly elevated in patients with PPSLD in both cohorts. (Figure 2B). Prealbumin levels were markedly lower in the PPSLD group in Cohort-1 (15.2 vs. 23.9 mg/dL, *p* < 0.0001). However, no significant difference (*p* = 0.057) was noted in Cohort-2 (Figure 2C), possibly reflecting the effect of nutritional counseling in the early postoperative phase.

Despite clinical features consistent with hepatic steatosis and biochemical evidence of liver injury, patients with PPSLD in both cohorts exhibited significantly higher serum total bile acid levels than those in the non-PPSLD group (33.9 vs. 7.4 μmol/L in Cohort-1, *p* = 0.0002; 31.4 vs. 15.1 μmol/L in Cohort-2 at 12 months postoperatively, *p* = 0.011; Figure 2D).

In both cohorts, low- and high-density lipoprotein cholesterol concentrations and apolipoprotein C2 levels were significantly lower in the PPSLD group, whereas triglyceride levels did not differ between the groups (Figure 2E).

Although body mass index (BMI) and body fat percentage were not significantly different in Cohort-1, patients with PPSLD showed poorer weight recovery and a significant reduction in skeletal muscle mass. No differences were observed in serum zinc levels or thyroid function (Table S3). These findings are consistent with a postoperative malnutrition-associated steatosis rather than an obesity-driven phenotype.

### *Selective expansion of* Lactobacillaceae *after pancreatic resection*

**Figure 3.**
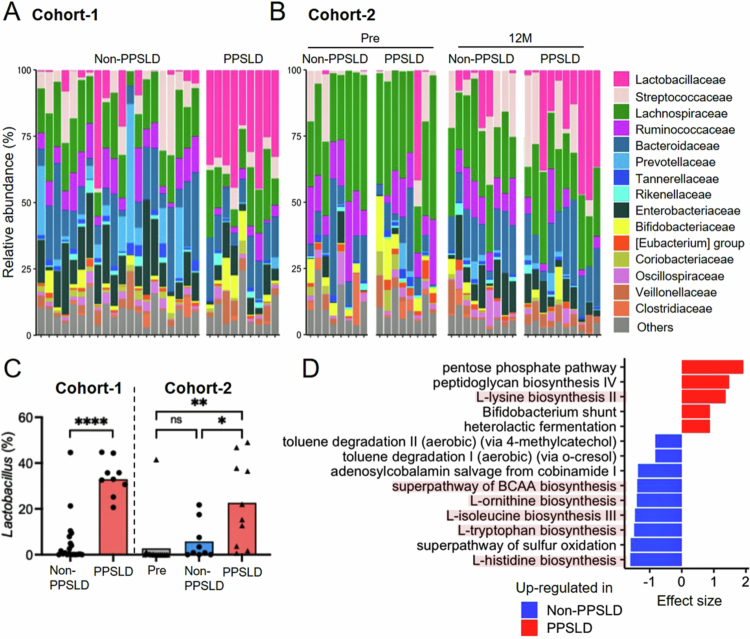
Significant increase in *Lactobacillus* abundance in patients with PPSLD. (A, B) Fecal microbiota at the family level in (A) Cohort-1 and (B) Cohort-2. (C) Abundance of *Lactobacillus* in Cohort-1 and Cohort-2. (D) Pathways upregulated in each group in Cohort-1. *Each dot represents one patient, and the bars indicate median values. Statistical significance was assessed using the Mann–Whitney *U* test in panels C. ^∗^*p* < 0.05; ^∗∗^*p* < 0.01; ^∗∗∗∗^*p* < 0.0001; ns, not significant.. PPSLD, post-pancreatectomy steatotic liver disease.

We examined stool samples from post-pancreatectomy patients because the gut microbiota is integral to bile acid metabolism (especially by deconjugating and converting bile acids into secondary forms). In Cohort-1, in which PPSLD persisted for extended durations (14–73 months postoperatively), the abundance of *Lactobacillaceae* (primarily *Lactobacillus* spp.) was significantly increased in the PPSLD group (Figure 3A, C; Figure S3). A similar increase in *Lactobacillus* abundance was noted in Cohort-2 at 12 months postoperatively compared to the preoperative levels (Figure 3B, C). Although *Lactobacillus* spp. varied among the patients, *Lactobacillus reuteri* was the most commonly identified (Figure S4). These data suggest that intestinal *Lactobacillus* expansion is associated with the early development and maintenance of PPSLD.

Functional predictions using PICRUSt2 in Cohort-1 indicated reduced predicted microbial pathways for the synthesis of the branched-chain amino acids such as tryptophan and histidine in PPSLD relative to non-PPSLD (Figure 3D). Although these predictions do not directly reflect host amino acid metabolism, the reduced microbial metabolic potential may contribute to altered luminal nutrient availability, which could be relevant to the hypoalbuminemia and muscle mass loss observed in PPSLD.

### Altered gut microbiota is associated with bile acid deconjugation and FXR–FGF19 downregulation

**Figure 4.**
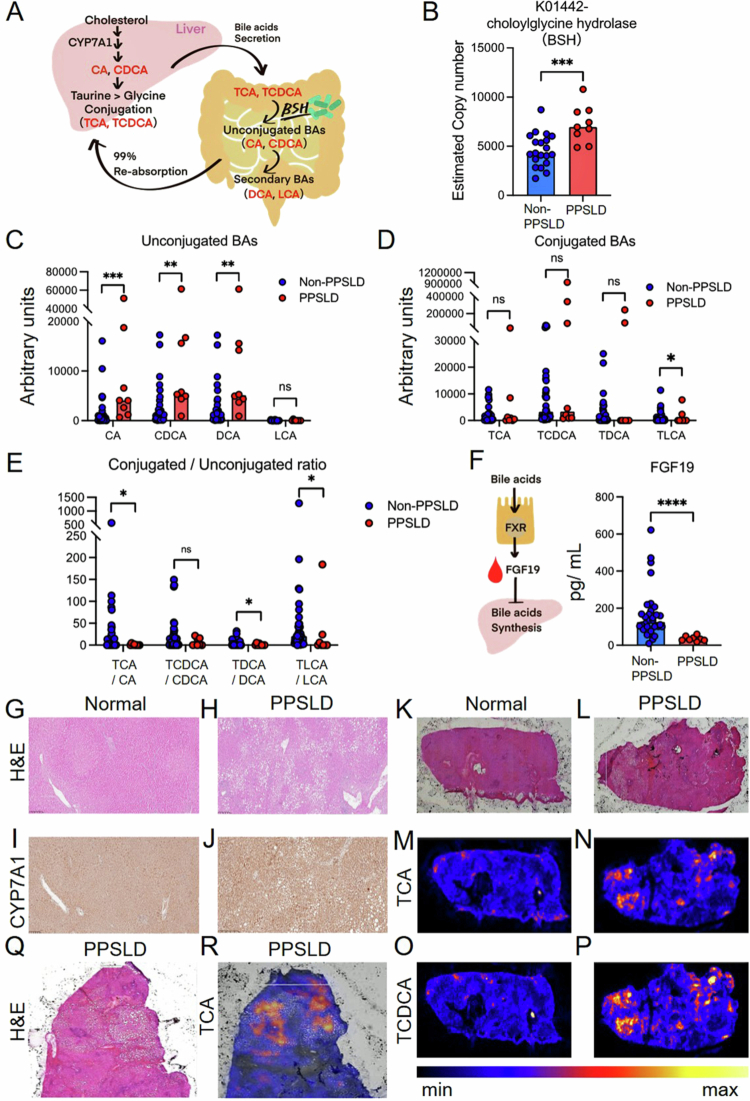
Altered bile acid profiles in patients with PPSLD. (A) The enterohepatic circulation of bile acids. (B) The functional prediction of bacteria was performed using PICRUSt2 analysis in Cohort-1. Estimated copy numbers of the choloylglycine hydrolase gene encoding BSH. (C–E) Serum bile acid composition in Cohort-1. (C) Unconjugated bile acid, (D) conjugated bile acid, and (E) conjugated-to-unconjugated ratio. (F) Serum FGF19 levels in Cohort-1. (G, H) Hematoxylin and eosin staining of (G) normal and (H) PPSLD livers (scale bar: 100 µm). (I, J) Immunohistochemical staining of hepatic CYP7A1 in (I) normal and (J) PPSLD livers (scale bar: 50 µm). (M–P) Bile acid imaging of TCA (M, N) and TCDCA (O, P) was performed by imaging mass spectrometry in (K, M, O) normal and (L, N, P) PPSLD livers. (Q, R) Enlarged views of PPSLD liver sections. (Q) Hematoxylin and eosin staining and (R) bile acid imaging of TCA. Each dot represents one patient, and the bar indicates the median values. Statistical significance was assessed using the Mann–Whitney *U* test (panels B–F). ^∗^*p* < 0.05; ^∗∗^*p* < 0.01; ^∗∗∗^*p* < 0.001; ^∗∗∗∗^*p* < 0.0001; ns, not significant. BSH, bile salt hydrolase; CA, cholic acid; CDCA, chenodeoxycholic acid; DCA, deoxycholic acid; FGF19, fibroblast growth factor 19; LCA, lithocholic acid; PPSLD, post-pancreatectomy steatotic liver disease; TCA, taurocholic acid; TCDCA, taurochenodeoxycholic acid; TDCA, taurodeoxycholic acid; TLCA, taurolithocholic acid.

The distinct gut microbial composition observed in patients with PPSLD is associated with differences in both total bile acid levels and bile acid composition. In humans, primary bile acids such as cholic acid (CA) and chenodeoxycholic acid (CDCA) are conjugated with glycine or taurine in the liver and stored in the gallbladder. Upon entering the small intestine, these conjugated bile acids are deconjugated by microbial bile salt hydrolase (BSH), followed by dehydroxylation into secondary bile acids such as deoxycholic acid (DCA), lithocholic acid (LCA), and UDCA (Figure 4A).

PICRUSt2 analysis revealed a significant increase in the predicted copy number of the choloylglycine hydrolase gene, which encodes BSH, in PPSLD (Figure 4B), suggesting increased microbial deconjugation potential in the PPSLD-associated microbiota. LC-MS assays in Cohort-1 revealed elevated levels of unconjugated CA, CDCA, and DCA in PPSLD (5.0- to 12.3-fold higher than in controls), whereas taurine-conjugated forms did not differ significantly (Figure 4C, D). The ratios of taurine-conjugated to unconjugated bile acids were significantly reduced for CA, DCA, and LCA in the PPSLD group (Figure 4E), indicating a disproportionate increase in deconjugated species.

Bile acids activate the FXR in intestinal epithelial cells, which induces FGF19 secretion into the portal circulation. Because deconjugated CA is a relatively weak FXR agonist, a shift toward a higher proportion of deconjugated CA may attenuate FXR activation.^9^ Consistent with this interpretation, serum FGF19 levels were lower in the PPSLD group in Cohort-1 (31.6 vs. 125.4 pg/mL, *p* = 0.01; Figure 4F).

Histological examination of a liver specimen from a single patient with PPSLD showed significant lipid droplet deposition, predominantly around the central vein, in the PPSLD samples (Figure 4G, H). Immunostaining of the same liver sample revealed increased expression of CYP7A1, aligning with the lower FGF19 levels and suggesting reduced feedback regulation of CYP7A1 and altered bile acid homeostasis (Figure 4I, J).^27^ Furthermore, TCA and TCDCA concentrations were elevated in PPSLD liver tissue, correlating with the regions of hepatic lipid accumulation (Figure 4M–R). These findings are consistent with impaired negative feedback regulation of CYP7A1 via FGF19, a pathway known to control bile acid synthesis.

To examine how anatomical loss relates to the biochemical and microbial abnormalities observed in PPSLD, we performed exploratory correlation analyzes. Remnant pancreas proportion showed significant correlations with liver attenuation (*ρ* = 0.38, *p* = 0.008), total serum bile acids (*ρ* = −0.60, *p* < 0.001), and *Lactobacillus* abundance (*ρ* = −0.43, *p* = 0.020). A positive trend was also observed for FGF19 levels (*ρ* = 0.26, *p* = 0.106). These associations suggest that smaller pancreatic remnants may be linked to bile-acid dysregulation and selective microbial shifts characteristic of PPSLD.

To further evaluate the relationship between microbial changes and enterohepatic signaling, we assessed the association between *Lactobacillus* abundance and circulating FGF19 in Cohort-1. A modest but significant inverse correlation was observed (Figure S5), supporting a potential link between increased *Lactobacillus* and attenuated FXR-FGF19 signaling, though causality cannot be inferred from this cross-sectional analysis.

### Depletion of nutritional metabolites related to lipid metabolism and protein catabolism in PPSLD

**Figure 5.**
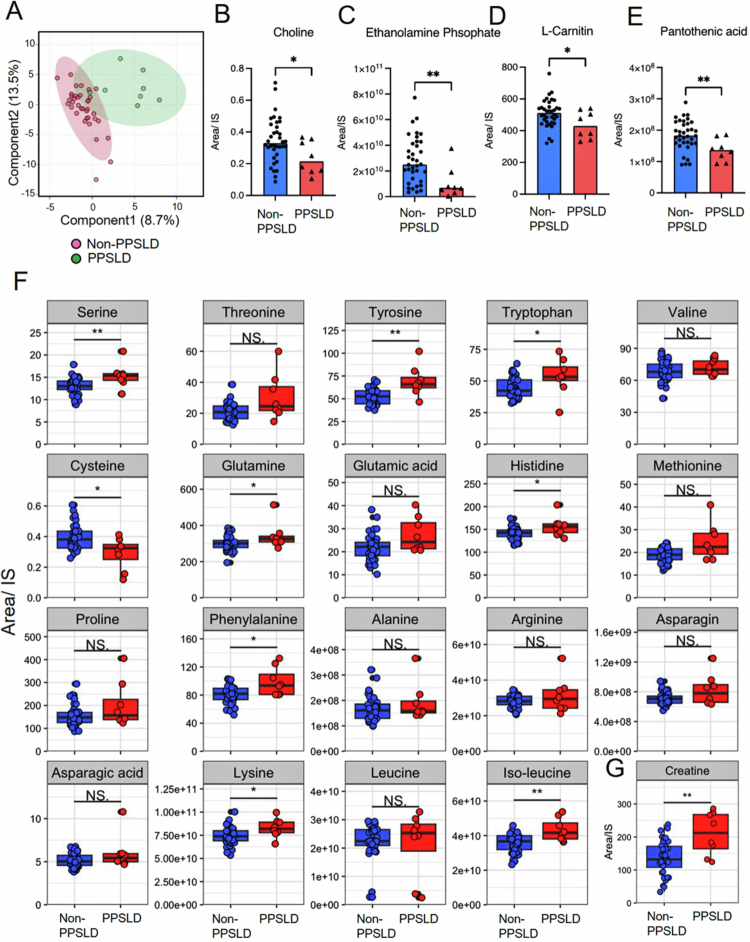
Altered metabolite profiles in sera of patients with PPSLD. (A–E) Serum metabolome analysis in Cohort-1. (A) PLSDA. (B–E) Serum levels of (B) choline, (C) ethanolamine phosphate, (D) L-carnitine, and (E) pantothenic acid. (F, G) Serum levels of (F) amino acids and (G) creatine. *Each dot represents a single patient, and bars indicate median values. Statistical significance was evaluated using the Mann–Whitney *U* test across all panels. ^∗^*p* < 0.05; ^**^p < 0.01; ns, not significant. *Glycine was not fully extracted or identified using the measurement protocol. PLSDA, partial least squares discriminant analysis; PPSLD, post-pancreatectomy steatotic liver disease.

In Cohort-1, patients with PPSLD exhibited a hypotrophic phenotype characterized by decreased serum lipid parameters and lower skeletal muscle mass despite adequate dietary intake (Table S4). Multivariate metabolomic analysis (PLS-DA) revealed distinct shifts in the levels of water-soluble metabolites (Figure 5A). The levels of choline and phosphoethanolamine, which are essential precursors of phosphatidylcholine, were significantly reduced (Figure 5B, C). Phosphatidylcholine is critical for packaging and transporting triglycerides via very low-density lipoproteins.^28^ This shortage may be exacerbated by poor lipid absorption, which is reflected in the high incidence of steatorrhea (Table 1).

Metabolites involved in fatty acid metabolism, such as carnitine and pantothenic acid, were similarly depleted (Figure 5D, E). Reduced carnitine can limit *β*-oxidation,^29^ whereas pantothenic acid deficiency compromises CoA synthesis, further hindering lipid oxidation and lipoprotein production. Interestingly, the levels of several amino acids (serine, tyrosine, tryptophan, glutamine, histidine, phenylalanine, lysine, and isoleucine) were elevated in PPSLD (Figure 5F), along with increased levels of serum creatine (Figure 5G) marker of muscle breakdown. These findings may indicate “protein starvation,” possibly stemming from decreased protease secretion, which leads to compensatory protein catabolism and muscle wasting.

## Discussion

This study is the first to characterize the pathophysiological features of PPSLD in relation to gut microbiome alterations and bile acid dysregulation. Our findings suggest a potential pathological sequence involving elevated deconjugated bile acids, especially CA, possibly driven by gut bacteria with high BSH activity, accompanied by the suppression of the FXR–FGF19 axis, and biochemical features consistent with upregulated hepatic bile acid synthesis. Concurrently, patients with PPSLD show reduced absorption of metabolites critical for phospholipid synthesis and fatty acid metabolism, indicating a multifactorial association with a metabolically impaired hepatic steatosis phenotype.

The marked shift in the gut microbiota of patients with PPSLD compared to that of individuals without PPSLD appears to be closely related to the altered gastrointestinal environment following pancreatic resection. We speculate that reduced bicarbonate secretion may lead to a lower intestinal pH,^30^ creating conditions that support *Lactobacillaceae* expansion, which thrive in mildly acidic and bile acid-rich niches, although we did not directly measure intestinal pH in this study. Comparable microbial shifts have also been reported in canine models of exocrine pancreatic insufficiency, where *Lactobacillaceae* and *Streptococcaceae* flourished under similar conditions.^31^ Notably, we observed more pronounced increases in *Lactobacillaceae* in patients with long-standing PPSLD, implying a stabilizing relationship between altered microbiota and disease over time.

Although PPSLD presents as hepatic steatosis, the underlying mechanisms differ sharply from those of MASLD. Obesity-driven MASLD is often associated with a reduced abundance of BSH-active gut bacteria, which can lower cholesterol levels in animal models.^32^ In contrast, PPSLD involves the overgrowth of BSH-active Lactobacillaceae, which is associated with higher bile acid turnover and reduced cholesterol levels. These opposing patterns of BSH activity underscore the intricate interplay between the gut microbiome, host metabolic status, and development of steatosis.

Central to bile acid-mediated communication between the liver and intestines is the FXR–FGF19 axis.^11^ Bile acids activate FXR, reducing hepatic lipogenesis and intestinal lipid absorption. In PPSLD, this regulatory axis appears suppressed, a pattern consistent with reduced feedback regulation of CYP7A1 and altered bile acid homeostasis. Changes in the bile acid composition may further influence FXR activity. Certain secondary bile acids, such as DCA, can act as FXR antagonists, whereas CA and UDCA are relatively inactive.^9^^,^^10^ In our patients with PPSLD, CA levels increased significantly (12-fold), overshadowing the fivefold increases in DCA and CDCA, suggesting a pronounced shift toward FXR-antagonistic or FXR-neutral bile acids. Elevated levels of deconjugated bile acids, which are more hydrophobic, may also reduce their efficient reabsorption via the apical sodium-dependent bile acid transporter,^33^ weakening enterohepatic circulation and further impairing the FXR–FGF19 axis. Additional secondary oxidation in the colon can generate higher cytotoxic DCA levels, imposing further stress on hepatic homeostasis.^32^

To further contextualize these findings within the clinical setting, we examined how pancreatic remnant volume relates to key microbial and biochemical markers. Remnant pancreas proportion was significantly correlated with liver attenuation, total serum bile acid levels, and *Lactobacillus* abundance, and showed a positive trend with FGF19 levels. These associations suggest that pancreatic volume loss—likely representing the degree of exocrine insufficiency—is closely linked to bile acid dysregulation and gut microbial alterations characteristic of PPSLD.

Because PPSLD did not occur after DP in our cohort, we were unable to evaluate the potential contribution of duodenal loss or alterations in upper gastrointestinal physiology—factors that are specific to PD. Conversely, although PPSLD occurred relatively frequently after TP, the number of cases was too small to isolate the effect of complete pancreatic loss. Within this overall context, the PD subgroup provided the clearest evidence: among PD patients, smaller remnant pancreatic volume was significantly associated with PPSLD. These findings suggest that, while procedure-specific anatomical changes may also play a role, the extent of pancreatic tissue loss remains a major determinant of PPSLD.

Although our study was not designed to evaluate the effects of chemotherapy, a higher proportion of PPSLD cases in Cohort-2 had received postoperative chemotherapy. In contrast, patients in Cohort-1 were sampled at a median of 31 months after surgery, well beyond the expected duration of adjuvant therapy, making persistent drug effects unlikely. To address this potential confounder within the limits of our dataset, exploratory comparisons in PD cases showed higher *Lactobacillus* abundance and a trend toward higher total bile acid levels among chemotherapy-exposed patients (Table S5). However, chemotherapy was not an independent predictor of PPSLD in multivariable analysis. These observations suggest that treatment-related factors may transiently modulate microbial or metabolic parameters, but their influence appears secondary to the degree of pancreatic remnant loss.

PERT remains the primary treatment for PPSLD.^34^ However, our observations suggest that PERT alone is often insufficient to prevent disease progression. Even with dietary management, patients frequently experience rapid loss of muscle and fat mass during the first three months postoperatively, with only limited improvement after one year. Therefore, correcting gut dysbiosis may offer a promising adjunctive strategy. Potential approaches include alkalinizing the intestinal environment to suppress *Lactobacillaceae* overgrowth and targeting bile acid metabolism via pharmacological agents such as FXR agonizts or FGF19 analogs to restore metabolic balance.

This study has several limitations. First, the relatively small sample size may limit the generalizability of our findings. Second, as an observational study, it cannot establish causality; thus, our findings should be interpreted as associations rather than mechanistic conclusions. Third, residual confounding cannot be fully excluded. Finally, hepatic analyzes were based on a single liver sample per group, limiting the robustness and reproducibility of tissue-level findings. Further validation in larger cohorts and the development of experimental models specific to PPSLD are warranted to better elucidate the underlying mechanisms.

In summary, PPSLD frequently affects patients with small pancreatic remnants following PD or TP. Our data suggest that gut dysbiosis—characterized by the expansion of *Lactobacillaceae*—may lead to excessive bile acid deconjugation, suppression of the FXR–FGF19 axis, impaired enterohepatic circulation, and malabsorption of key lipid-related metabolites. Based on these associations, we propose that future studies explore (1) early intensification of PERT to mitigate exocrine insufficiency and potential dysbiosis, (2) supplementation with choline and carnitine to improve nutrient deficits, and (3) targeted modulation of the intestinal environment or FXR–FGF19 signaling as a therapeutic strategy.

## Supplementary Material

Supplementary material Supplementary Material.

## Data Availability

All sequences and supplementary files associated with this study are available in the NCBI Sequence Read Archive (SRA) under BioProject ID PRJNA1226396 (https://www.ncbi.nlm.nih.gov/sra/PRJNA1226396).

## List of abbreviations

AST: aspartate aminotransferase
BMI: body mass index
BSH: bile salt hydrolase
CA: cholic acid
CDCA: chenodeoxycholic acid
CT: computed tomography
DCA: deoxycholic acid
DP: distal pancreatectomy
ESI: electrospray ionization
FGF19: fibroblast growth factor 19
FIB-4: fibrosis-4 index
FXR: farnesoid X receptor
LCA: lithocholic acid
LC/MS: liquid chromatography-mass spectrometry
LC-MS/MS: liquid chromatography-tandem mass spectrometry
MASLD: metabolic dysfunction-associated steatotic liver disease
MRM: multiple reaction monitoring
PD: pancreaticoduodenectomy
PDAC: pancreatic ductal adenocarcinoma
PERT: pancreatic enzyme replacement therapy
PPSLD: post-pancreatectomy steatotic liver disease
ROI: region of interest
TP: total pancreatectomy
UDCA: ursodeoxycholic acid

## References

[1] Doi R, Imamura M, Hosotani R, Imaizumi T, Hatori T, Takasaki K, Funakoshi A, Wakasugi H, Asano T, Hishinuma S, et al. Surgery versus radiochemotherapy for resectable locally invasive pancreatic cancer: final results of a randomized multi-institutional trial. Surg Today. 2008;38(11):1021–1028. doi: 10.1007/s00595-007-3745-8. 18958561

[2] Kato H, Isaji S, Azumi Y, Kishiwada M, Hamada T, Mizuno S, Usui M, Sakurai H, Tabata M. Development of nonalcoholic fatty liver disease (NAFLD) and nonalcoholic steatohepatitis (NASH) after pancreaticoduodenectomy: proposal of a postoperative NAFLD scoring system. J Hepatobiliary Pancreat Sci. 2010;17(3):296–304. doi: 10.1007/s00534-009-0187-2. 19809782

[3] Yasukawa K, Shimizu A, Yokoyama T, Kubota K, Notake T, Seki H, Kobayashi A, Soejima Y. Preventive effect of high-dose digestive enzyme management on development of nonalcoholic fatty liver disease after pancreaticoduodenectomy: a randomized controlled clinical trial. J Am Coll Surg. 2020;231(6):658–669. doi: 10.1016/j.jamcollsurg.2020.08.761. 32927075

[4] Miura H, Ijichi M, Ando Y, Hayama K, IIhara K, Yamada H, Bandai Y. A rapidly progressive and fatal case of nonalcoholic steatohepatitis following pancreaticoduodenectomy. Clin J Gastroenterol. 2013;6(6):470–475. doi: 10.1007/s12328-013-0421-y. 26182139

[5] Yu HH, Shan YS, Lin PW. Effect of pancreaticoduodenectomy on the course of hepatic steatosis. World J Surg. 2010;34(9):2122–2127. doi: 10.1007/s00268-010-0636-8. 20502896

[6] Le Roy T, Llopis M, Lepage P, Bruneau A, Rabot S, Bevilacqua C, Martin P, Philippe C, Walker F, Bado A, et al. Intestinal microbiota determines development of non-alcoholic fatty liver disease in mice. Gut. 2013;62(12):1787–1794. doi: 10.1136/gutjnl-2012-303816. 23197411

[7] Boursier J, Mueller O, Barret M, Machado M, Fizanne L, Araujo-Perez F, Guy CD, Seed PC, Rawls JF, David LA, et al. The severity of nonalcoholic fatty liver disease is associated with gut dysbiosis and shift in the metabolic function of the gut microbiota. Hepatology. 2016;63(3):764–775. doi: 10.1002/hep.28356. 26600078 PMC4975935

[8] Aron-Wisnewsky J, Vigliotti C, Witjes J, Le P, Holleboom AG, Verheij J, Nieuwdorp M, Clément K. Gut microbiota and human NAFLD: disentangling microbial signatures from metabolic disorders. Nat Rev Gastroenterol Hepatol. 2020;17(5):279–297. doi: 10.1038/s41575-020-0269-9. 32152478

[9] Parks DJ, Blanchard SG, Bledsoe RK, Chandra G, Consler TG, Kliewer SA, Stimmel JB, Willson TM, Zavacki AM, Moore DD, et al. Bile acids: natural ligands for an orphan nuclear receptor. Sci. 1999;284(5418):1365–1368. doi: 10.1126/science.284.5418.1365. 10334993

[10] Jiao N, Baker SS, Chapa-Rodriguez A, Liu W, Nugent CA, Tsompana M, Mastrandrea L, Buck MJ, Baker RD, Genco RJ, et al. Suppressed hepatic bile acid signalling despite elevated production of primary and secondary bile acids in NAFLD. Gut. 2018;67(10):1881–1891. doi: 10.1136/gutjnl-2017-314307. 28774887

[11] Clifford BL, Sedgeman LR, Williams KJ, Morand P, Cheng A, Jarrett KE, Chan AP, Brearley-Sholto MC, Wahlström A, Ashby JW, et al. FXR activation protects against NAFLD via bile-acid-dependent reductions in lipid absorption. Cell Metab. 2021;33(8):1671–1684. e4. doi: 10.1016/j.cmet.2021.06.012. 34270928 PMC8353952

[12] Kodama Y, Ng CS, Wu TT, Ayers GD, Curley SA, Abdalla EK, Vauthey JN, Charnsangavej C. Comparison of CT methods for determining the fat content of the liver. AJR Am J Roentgenol. 2007;188(5):1307–1312. doi: 10.2214/AJR.06.0992. 17449775

[13] Ma X, Holalkere NS, Kambadakone RA, Mino-Kenudson M, Hahn PF, Sahani DV. Imaging-based quantification of hepatic fat: methods and clinical applications. Radiographics. 2009;29(5):1253–1277. doi: 10.1148/rg.295085186. 19755595

[14] Miyamoto R, Takahashi A, Ogasawara A, Ogura T, Kitamura K, Ishida H, Matsudaira S, Nozu S, Kawashima Y. Three-dimensional simulation of the pancreatic parenchyma, pancreatic duct and vascular arrangement in pancreatic surgery using a deep learning algorithm. PLoS One. 2022;17(10):e0276600. doi: 10.1371/journal.pone.0276600. 36306322 PMC9616217

[15] Sterling RK, Lissen E, Clumeck N, Sola R, Correa MC, Montaner J, S Sulkowski M, Torriani FJ, Dieterich DT, Thomas DL, et al. Development of a simple noninvasive index to predict significant fibrosis in patients with HIV/HCV coinfection. Hepatology. 2006;43(6):1317–1325. doi: 10.1002/hep.21178. 16729309

[16] Kato K, Isaji S, Kawarada Y, Hibasami H, Nakashima K. Effect of zinc administration on pancreatic regeneration after 80% pancreatectomy. Pancreas. 1997;14(2):158–165. doi: 10.1097/00006676-199703000-00008. 9057188

[17] Takahashi K, Yoshimura Y, Kaimoto T, Kunii D, Komatsu T, Yamamoto S. Validation of a food frequency questionnaire based on food groups for estimating individual nutrient intake. Jpn J Nutr Diet. 2001;59(5):221–232. doi: 10.5264/eiyogakuzashi.59.221.

[18] Takahashi S, Tomita J, Nishioka K, Hisada T, Nishijima M. Development of a prokaryotic universal primer for simultaneous analysis of *Bacteria* and *Archaea* using next-generation sequencing. PLoS One. 2014;9(8):e105592. doi: 10.1371/journal.pone.0105592. 25144201 PMC4140814

[19] Hisada T, Endoh K, Kuriki K. Inter- and intra-individual variations in seasonal and daily stabilities of the human gut microbiota in Japanese. Arch Microbiol. 2015;197(7):919–934. doi: 10.1007/s00203-015-1125-0. 26068535 PMC4536265

[20] Bolyen E, Rideout JR, Dillon MR, Bokulich NA, Abnet CC, Al-Ghalith GA, Alexander H, Alm EJ, Arumugam M, Asnicar F, et al. Author correction: reproducible, interactive, scalable and extensible microbiome data science using QIIME 2. Nat Biotechnol. 2019;37(9):1091–1091. doi: 10.1038/s41587-019-0252-6. 31399723

[21] Callahan BJ, McMurdie PJ, Rosen MJ, Han AW, Johnson AJ, Holmes SP. DADA2: High-resolution sample inference from Illumina amplicon data. Nat Methods. 2016;13(7):581–583. doi: 10.1038/nmeth.3869. 27214047 PMC4927377

[22] Pruesse E, Quast C, Knittel K, Fuchs BM, Ludwig W, Peplies J, Glöckner FO. SILVA: a comprehensive online resource for quality checked and aligned ribosomal RNA sequence data compatible with ARB. Nucleic Acids Res. 2007;35(21):7188–7196. doi: 10.1093/nar/gkm864. 17947321 PMC2175337

[23] Camacho C, Coulouris G, Avagyan V, Ma N, Papadopoulos J, Bealer K, Madden TL. BLAST+: architecture and applications. BMC Bioinform. 2009;10:421. doi: 10.1186/1471-2105-10-421. PMC2803857 20003500

[24] Weiss S, Xu ZZ, Peddada S, Amir A, Bittinger K, Gonzalez A, Lozupone C, Zaneveld JR, Vázquez-Baeza Y, Birmingham A, et al. Normalization and microbial differential abundance strategies depend upon data characteristics. Microbiome. 2017;5(1):27. doi: 10.1186/s40168-017-0237-y. 28253908 PMC5335496

[25] Zhang B, Vogelzang A, Miyajima M, Sugiura Y, Wu Y, Chamoto K, Nakano R, Hatae R, Menzies RJ, Sonomura K, et al. B cell-derived GABA elicits IL-10^+^ macrophages to limit anti-tumour immunity. Natur. 2021;599(7885):471–476. doi: 10.1038/s41586-021-04082-1. PMC8599023 34732892

[26] Bassi C, Marchegiani G, Dervenis C, Sarr M, Abu Hilal M, Adham M, Allen P, Andersson R, Asbun HJ, Besselink MG, et al. The 2016 update of the international study group (ISGPS) definition and grading of postoperative pancreatic fistula: 11 years after. Surgery. 2017;161(3):584–591. doi: 10.1016/j.surg.2016.11.014. 28040257

[27] Inagaki T, Choi M, Moschetta A, Peng L, Cummins CL, McDonald JG, Luo G, Jones SA, Goodwin B, Richardson JA, et al. Fibroblast growth factor 15 functions as an enterohepatic signal to regulate bile acid homeostasis. Cell Metab. 2005;2(4):217–225. doi: 10.1016/j.cmet.2005.09.001. 16213224

[28] Yao ZM, Vance DE. The active synthesis of phosphatidylcholine is required for very low density lipoprotein secretion from rat hepatocytes. J Biol Chem. 1988;263(6):2998–3004. doi: 10.1016/S0021-9258(18)69166-5. 3343237

[29] Savic D, Hodson L, Neubauer S, Pavlides M. The importance of the fatty acid transporter L-carnitine in non-alcoholic fatty liver disease (NAFLD). Nutrients. 2020;12(8):2178. doi: 10.3390/nu12082178. 32708036 PMC7469009

[30] Dutta SK, Russell RM, Iber FL. Influence of exocrine pancreatic insufficiency on the intraluminal pH of the proximal small intestine. Dig Dis Sci. 1979;24(7):529–534. doi: 10.1007/BF01489321. 37059

[31] Isaiah A, Parambeth JC, Steiner JM, Lidbury JA, Suchodolski JS. The fecal microbiome of dogs with exocrine pancreatic insufficiency. Anaerobe. 2017;45:50–58. doi: 10.1016/j.anaerobe.2017.02.010. 28223257

[32] Choi SB, Lew LC, Yeo SK, Nair Parvathy S, Liong MT. Probiotics and the BSH-related cholesterol lowering mechanism: a Jekyll and Hyde scenario. Crit Rev Biotechnol. 2015;35(3):392–401. doi: 10.3109/07388551.2014.889077. 24575869

[33] Winston JA, Theriot CM. Diversification of host bile acids by members of the gut microbiota. Gut Microbes. 2020;11(2):158–171. doi: 10.1080/19490976.2019.1674124. 31595814 PMC7053883

[34] Whitcomb DC, Lehman GA, Vasileva G, Malecka-Panas E, Gubergrits N, Shen Y, Sander-Struckmeier S, Caras S. Pancrelipase delayed-release capsules (CREON) for exocrine pancreatic insufficiency due to chronic pancreatitis or pancreatic surgery: a double-blind randomized trial. Am J Gastroenterol. 2010;105(10):2276–2286. doi: 10.1038/ajg.2010.201. 20502447

