# Supplementary material for: Increased intestinal Lactobacillus abundance in post-pancreatectomy steatotic liver disease is associated with altered bile acid metabolism and FXR–FGF19 pathway suppression
Source: Gut Microbes Rep. 2025 Dec 27;3(1):2607927. doi: 10.1080/29933935.2025.2607927 (PMC12938879; doi:10.1080/29933935.2025.2607927)
Supplement: Supplementary material [file KGMR_A_2607927_SM5877.zip › Supplementary Table 1.docx]

**Supplementary Table 1. Longitudinal changes in serum and body composition parameters over one year in Cohort-2**

| **Cohort-2** | **Parameters** | | **Pre (n=25)** | **12M (n=22)** | **P value** |  |
| --- | --- | --- | --- | --- | --- | --- |
|  |  |  |  |  |  |  |
| Liver-related | TBA (μmol/L) | | 4.2 [1.0-27.5] | 19.2 [3.3-74.9] | **<0.0001*** |  |
|  | AST (U/L) | | 20 [14-51] | 26 [16-73] | **0.01*** |  |
|  | ALT (U/L) | | 18 [9-73] | 26 [12-69] | 0.07 |  |
|  | Γ-GTP (IU/L) | | 31 [10-254] | 28 [14-103] | 0.86 |  |
|  | FIB-4 index | | 1.58 [0.52-2.44] | 1.82 [0.64-3.71] | **0.04*** |  |
| Protein synthesis | Total protein (g/dL) | | 6.5 [5.7-7.6] | 6.9 [5.9-7.8] | **0.001*** |  |
|  | Albumin (g/dL) | | 4.0 [3.5-4.7] | 4.2 [3.2-4.7] | 0.24 |  |
|  | Retinol-binding protein (mg/dL) | | 2.5 [1.5-3.9] | 2.3 [1.2-4.6] | 0.66 |  |
|  | Prealbumin (mg/dL) | | 20.2 [14.4-31.5] | 21.3 [11.7-35.2] | 0.3 |  |
| Carbohydrate | Hemoglobin A1c (%) | | 5.9 [5.3-8.4] | 6.3 [5.0-9.9] | 0.07 |  |
| Lipid metabolism | Cholesterol (mg/dL) | T-cho | 190 [119-252] | 158 [86-231] | **0.0007*** |  |
|  |  | LDL-cho | 114 [38-160] | 82 [35-153] | **0.0002*** |  |
|  |  | HDL-cho | 66 [37-87] | 64 [32-101] | 0.83 |  |
|  | Triglyceride (mg/dL) | | 85 [47-271] | 72 [46-201] | **0.007*** |  |
|  | Free fatty acid (μEq/L) | | 528 [84-1834] | 602 [166-1280] | 0.47 |  |
|  | Apolipoprotein（mg/dL） | apoB | 82 [30-118] | 76 [35-104] | **0.003*** |  |
|  |  | apoE | 4.2 [2.0-7.4] | 2.9 [1.9-7.0] | **<0.0001*** |  |
|  |  | apoC2 | 3.7 [1.2-6.9] | 2.5 [1.1-3.8] | **0.0004*** |  |
| Body composition | BMI (kg/m^2^) | | 21.8 [18.6-28.9] | 19.4 [17.0-27.0] | **<0.0001*** |  |
|  | Body weight gain ratio | | N.A. | 0.90 [0.78-1.02] | N.A. |  |
|  | Skeletal muscle area (cm^2^/m^2^) | | 45.4 [31.5-67.5] | 43.6 [32.4-62.2] | **0.04*** |  |
|  | Iliopsoas muscle area (cm^2^/m^2^) | | 7.1 [4.3-11.2] | 6.3 [3.3-10.8] | **0.006*** |  |
|  | Body fat percentage (%) | | 30.4 [17.4-38.7] | 20.4 [5.0-35.0] | **<0.0001*** |  |
| Others | Zinc (μg/dL) | | 83.0 [65.0-124.0] | 85.0 [59.0-151.0] | 0.71 |  |
|  | fT3 (pg/mL) | | 3.03 [2.37-3.72] | 2.60 [1.77-3.18] | **<0.0001*** |  |
|  | fT4 (ng/dL) | | 1.05 [0.70-1.39] | 0.97 [0.71-1.30] | **0.02*** |  |
|  | TSH (μIU/mL) | | 1.73 [0.69-6.75] | 2.27 [0.02-12.61] | **0.01*** |  |

*Total bile acid levels increased significantly at 12 months (M) postoperatively. Prealbumin levels had returned to preoperative levels by 12M. LDL cholesterol and triglycerides were significantly reduced at 12M. BMI, skeletal muscle mass, psoas muscle mass, and body fat percentage were all significantly lower at 12M and had not returned to preoperative levels at 12M after pancreatic resection. Apolipoproteins showed a significant decrease from the preoperative period to 12M postoperatively.

^*^FIB-4 index can be calculated by the following formula: age x AST/platelet count [x 10^3^/μL] x (ALT)^1/2^[1].

^*^Body weight gain ratio was calculated by dividing the weight at 12M by the preoperative weight.

TBA, total bile acid; AST, aspartate aminotransferase; ALT, alanine aminotransferase; γ-GTP, γ-glutamyltranspeptidase; T-cho, total cholesterol; LDL-cho, low-density lipoprotein cholesterol; HDL-cho, high-density lipoprotein cholesterol; BMI, body mass index; fT3, free triiodothyronine; fT4, free thyroxine; TSH, thyroid stimulating hormone.

**Reference**

[1] Sterling RK, Lissen E, Clumeck N, Sola R, Correa MC, Montaner J, et al. Development of a simple noninvasive index to predict significant fibrosis in patients with HIV/HCV coinfection. Hepatology. 2006;43:1317-25.
