# Supplementary material for: Increased intestinal Lactobacillus abundance in post-pancreatectomy steatotic liver disease is associated with altered bile acid metabolism and FXR–FGF19 pathway suppression
Source: Gut Microbes Rep. 2025 Dec 27;3(1):2607927. doi: 10.1080/29933935.2025.2607927 (PMC12938879; doi:10.1080/29933935.2025.2607927)
Supplement: Supplementary material [file KGMR_A_2607927_SM5877.zip › Supplementary Table 2.docx]

**Supplementary Table 2. Multivariable logistic regression for PPSLD (Cohort-1, PD only)**

| **Parameters** | **aOR** | **95% CI** | **P value** |
| --- | --- | --- | --- |
| Age | 0.56 | 0.15 – 2.02 | 0.373 |
| Sex (male *vs* female) | 3.11 | 0.40 – 24.36 | 0.280 |
| BMI | 0.29 | 0.03 – 2.81 | 0.285 |
| Chemotherapy (yes *vs* no) | 3.69 | 0.30 – 44.70 | 0.305 |
| Remnant pancreas | 0.54 | 0.30 – 0.98 | **0.044*** |

Outcome: PPSLD (yes/no). Multivariable logistic regression model including age, sex, BMI, chemotherapy, and remnant pancreas proportion. Surgical subset: PD only.

Age (per 10 years), BMI (per 5 kg/m²), Remnant pancreas (per 5%-point increase).

PPSLD, post-pancreatectomy steatotic liver disease; PD, pancreaticoduodenectomy; aOR, adjusted odds ratio; CI, confidence interval; BMI, body mass index.
