# Supplementary material for: Increased intestinal Lactobacillus abundance in post-pancreatectomy steatotic liver disease is associated with altered bile acid metabolism and FXR–FGF19 pathway suppression
Source: Gut Microbes Rep. 2025 Dec 27;3(1):2607927. doi: 10.1080/29933935.2025.2607927 (PMC12938879; doi:10.1080/29933935.2025.2607927)
Supplement: Supplementary material [file KGMR_A_2607927_SM5877.zip › Supplementary Table 3.docx]

**Supplementary Table 3. Differences in Serum and Body Composition Parameters Based on the Presence or Absence of PPSLD in Cohort-1**

| **Cohort-1** | **Parameters** | | **Non-PPSLD (n=38)** | **PPSLD (n=9)** | **P value** |
| --- | --- | --- | --- | --- | --- |
| Liver-related | TBA (μmol/L) | | 7.4 [1.7-53.9] | 33.9 [13.1-69.4] | **0.0002*** |
|  | AST (U/L) | | 24 [13-42] | 39 [21-120] | **<0.0001*** |
|  | ALT (U/L) | | 19 [8-59] | 30 [21-126] | **0.0006*** |
|  | Γ-GTP (IU/L) | | 18 [9-84] | 16 [14-240] | 0.21 |
|  | FIB-4 index | | 1.66 [0.37-3.24] | 2.39 [1.15-3.93] | **0.002*** |
| Protein synthesis | Total protein (g/dL) | | 7.2 [6.1-8.2] | 6.9 [6.5-7.9] | **0.048*** |
|  | Albumin (g/dL) | | 4.4 [3.7-4.9] | 4.0 [3.6-4.3] | **0.0003*** |
|  | Retinol-binding protein (mg/dL) | | 2.8 [1.3-4.4] | 1.7 [1.4-2.8] | **0.0005*** |
|  | Prealbumin (mg/dL) | | 23.9 [15.7-37.0] | 15.2 [13.0-20.2] | **<0.0001*** |
| Carbohydrate | Hemoglobin A1c (%) | | 5.9 [4.8-7.9] | 6.1 [5.2-8.5] | 0.65 |
| Lipid metabolism | Cholesterol (mg/dL) | T-cho | 185 [115-317] | 135 [71-177] | **0.0001*** |
|  |  | LDL-cho | 104.5 [56-210] | 88 [39-108] | **0.01*** |
|  |  | HDL-cho | 63.5 [35-109] | 40 [17-67] | **<0.0001*** |
|  | Triglyceride (mg/dL) | | 87 [39-160] | 73 [57-108] | 0.53 |
|  | Free fatty acid (μEq/L) | | 650 [113-1218] | 560 [132-877] | 0.18 |
|  | Apolipoprotein（mg/dL） | apoB | 78.5 [47-154] | 71 [47-89] | 0.06 |
|  |  | apoE | 3.4 [1.6-5.9] | 2.7 [1.8-3.1] | **0.03*** |
|  |  | apoC2 | 3 [1.1-6.8] | 1.4 [0.9-2.6] | **0.003*** |
| Body composition | BMI (kg/m^2^) | | 20.5 [14.6-34.1] | 20.3 [18.3-23.7] | 0.86 |
|  | Body weight gain ratio | | 0.96 [0.75-1.13] | 0.88 [0.72-1.03] | **0.009*** |
|  | Skeletal muscle area (cm^2^/m^2^) | | 45.0 [31.8-60.6] | 41.7 [30.4-46.2] | **0.04*** |
|  | Iliopsoas muscle area (cm^2^/m^2^) | | 6.9 [3.7-10.4] | 6.1 [4.9-7.2] | 0.17 |
|  | Body fat percentage (%) | | 24.4 [9.1-41.7] | 22.4 [13.3-32.7] | 0.81 |
| Others | Zinc (μg/dL) | | 90 [53-129] | 86 [73-131] | 0.78 |
|  | fT3 (pg/mL) | | 3.00 [2.44-4.21] | 2.99 [2.75-3.46] | 0.93 |
|  | fT4 (ng/dL) | | 1.07 [0.74-1.49] | 0.99 [0.87-1.37] | 0.54 |
|  | TSH (μIU/mL) | | 2.32 [0.60-10.28] | 1.17 [0.56-3.41] | 0.11 |

^*^FIB-4 index can be calculated by the following formula: age x AST/platelet count [x 10^3^/μL] x (ALT)^1/2^ [1].

^*^Body weight gain ratio was calculated by dividing the current weight by the preoperative weight.

PPSLD; post-pancreatectomy steatotic liver disease; TBA, total bile acid; AST, aspartate aminotransferase; ALT, alanine aminotransferase; γ-GTP, γ-glutamyltranspeptidase; T-cho, total cholesterol; LDL-cho, low-density lipoprotein cholesterol; HDL-cho, high-density lipoprotein cholesterol; BMI, body mass index; fT3, free triiodothyronine; fT4, free thyroxine; TSH, thyroid stimulating hormone.

**Reference**

[1] Sterling RK, Lissen E, Clumeck N, Sola R, Correa MC, Montaner J, et al. Development of a simple noninvasive index to predict significant fibrosis in patients with HIV/HCV coinfection. Hepatology. 2006;43:1317-25.
