# Supplementary material for: Increased intestinal Lactobacillus abundance in post-pancreatectomy steatotic liver disease is associated with altered bile acid metabolism and FXR–FGF19 pathway suppression
Source: Gut Microbes Rep. 2025 Dec 27;3(1):2607927. doi: 10.1080/29933935.2025.2607927 (PMC12938879; doi:10.1080/29933935.2025.2607927)
Supplement: Supplementary material [file KGMR_A_2607927_SM5877.zip › Supplementary Table 4.docx]

**Supplementary Table 4. Dietary composition in the PPSLD group in Cohort-1**

| **Parameters** | **PPSLD (n=9)** | **sufficiency ratio (%)** |
| --- | --- | --- |
| Calorie intake (kcal/kg (IBW)) | 31.0 [23.0-47.1] | 84.9 [69.6-128.3] |
| Protein (g/kg (IBW)) | 1.3 [0.8-2.0] | 97.3 [67.2-133.0] |
| Fat (%) | 27.9 [14.6-32.4] | 82.0 [58.0-114.1] |
| Carbohydrate (%) | 56.0 [50.5-68.6] | 83.1 [65.5-161.1] |
| Fiber intake (g) | 12.0 [9.0-17.5] | 57.7 [42.6-92.4] |
| Saturated fatty acids (g) | 15.0 [13.5-21.9] | 81.0 [78.5-122.7] |
| Unsaturated fatty acids (g) | 26.7 [16.6-44.9] | 78.7 [47.6-123.5] |

*PPSLD patients received 31 kcal/kg (ideal body weight; IBW) of energy with adequate protein and fat intake from their diet. Nutritional sufficiency ratios compared with data from the "National Health and Nutrition Survey Japan 2019" showed that although fiber intake was low, PPSLD patients maintained a good balance of the three main nutrients, with no excessive intake of either saturated or unsaturated fatty acids.

* Sufficiency ratios were calculated by comparing them with data from the National Health and Nutrition Survey Japan 2019 for the general Japanese population.

PPSLD; post-pancreatectomy steatotic liver disease; IBW, ideal body weight
