# Supplementary material for: Increased intestinal Lactobacillus abundance in post-pancreatectomy steatotic liver disease is associated with altered bile acid metabolism and FXR–FGF19 pathway suppression
Source: Gut Microbes Rep. 2025 Dec 27;3(1):2607927. doi: 10.1080/29933935.2025.2607927 (PMC12938879; doi:10.1080/29933935.2025.2607927)
Supplement: Supplementary material [file KGMR_A_2607927_SM5877.zip › Supplementary Table 5.docx]

**Supplementary Table 5. Comparison of bile acids and *Lactobacillus* between chemotherapy groups (Cohort-1, PD only)**

| **Parameters** | **Chemo** | **Non-chemo** | **U** | **P value** |
| --- | --- | --- | --- | --- |
| Total bile acids (µmol/L) | 24.00 (36.0) | 20.05 (23.0) | 172.0 | 0.088 |
| Lactobacillus (relative abundance) | 8.76 (16.67) | 0.010 (0.12) | 36.0 | **0.003*** |

Non-parametric comparison (Mann–Whitney U test) between chemotherapy (Chemo) and non-chemotherapy (Non-chemo) groups in PD subset of Cohort-1. Data are shown as median (IQR).

PD: pancreaticoduodenectomy. P value<0.05 was considered statistically significant.
