# Supplementary material for: Increased intestinal Lactobacillus abundance in post-pancreatectomy steatotic liver disease is associated with altered bile acid metabolism and FXR–FGF19 pathway suppression
Source: Gut Microbes Rep. 2025 Dec 27;3(1):2607927. doi: 10.1080/29933935.2025.2607927 (PMC12938879; doi:10.1080/29933935.2025.2607927)
Supplement: Supplementary material [file KGMR_A_2607927_SM5877.zip › Supplementary_Figure_Legends.docx]

**Supplemental Figures；Figure legend**

**Supplementary Figure 1. Chart-flow of selection of candidates in Cohort-1 and 2**

PPSLD, post-pancreatectomy steatotic liver disease; PD, pancreaticoduodenectomy; TP, total pancreatectomy

**Supplementary Figure 2. Longitudinal changes in liver attenuation and 12-month changes (ΔHU) in Cohort-2.**

Paired scatter plots show individual trajectories of liver CT attenuation (HU) from baseline (Pre) to 12 months (12M) after pancreatectomy in Cohort-2, stratified by the development of PPSLD. Colored dots represent individual patients connected by lines to illustrate within-patient changes. The right subpanels display the 12-month change in HU (ΔHU, 12M – Pre) with group means ± SD. Liver attenuation remained stable in non-PPSLD patients, whereas those who developed PPSLD demonstrated a marked decline over 12 months.

PPSLD, post-pancreatectomy steatotic liver disease

**Supplementary Figure 3. Significant increase in *Lactobacillus* in PPSLD patients**

The fecal microbiota at the genus level (A) in Cohort-1 and (B) in Cohort-2.

PPSLD, post-pancreatectomy steatotic liver disease

**Supplementary Figure 4. Different *Lactobacillus* species are increased depending on the patient.**

The fecal microbiota at the species level (A) in Cohort-1 and (B) in Cohort-2.

PPSLD, post-pancreatectomy steatotic liver disease

**Supplementary Figure 5. Correlation between serum FGF19 levels and fecal *Lactobacillus* abundance.**

Pearson’s correlation analysis was performed using combined data from Cohorts 1 and 2 to examine the relationship between serum FGF19 concentrations (pg/ml) and the relative abundance (%) of fecal *Lactobacillus*. A significant negative correlation was observed. The grey shading represents the 95% confidence interval of the regression line.
