# Supplementary figures and images for: Increased intestinal Lactobacillus abundance in post-pancreatectomy steatotic liver disease is associated with altered bile acid metabolism and FXR–FGF19 pathway suppression
Source: Gut Microbes Rep. 2025 Dec 27;3(1):2607927. doi: 10.1080/29933935.2025.2607927 (PMC12938879; doi:10.1080/29933935.2025.2607927)

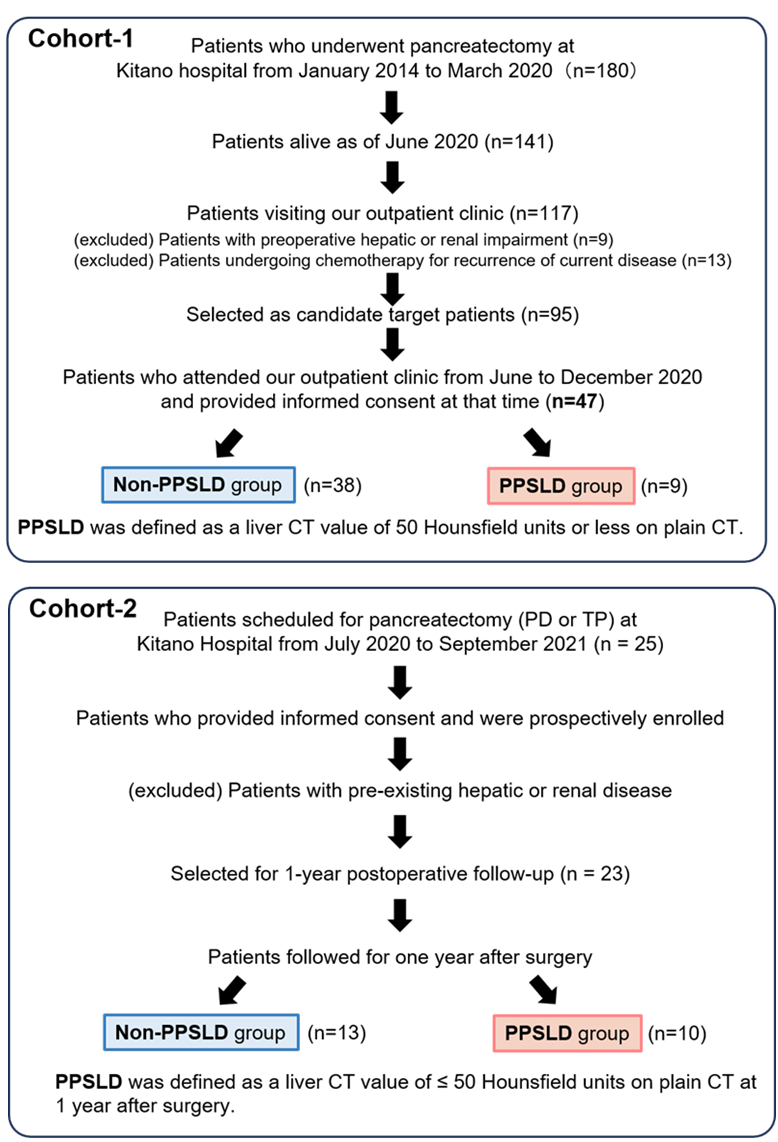

Supplement: Supplementary material [file KGMR_A_2607927_SM5877.zip › Fig S1.tif]

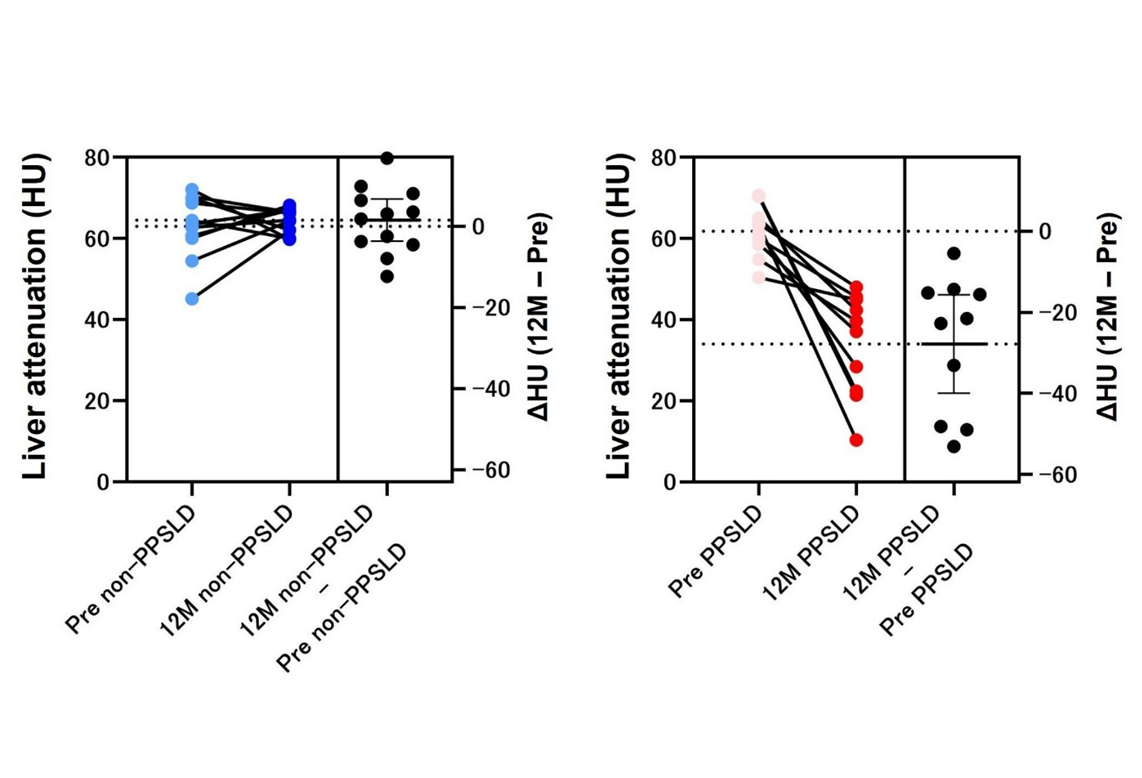

Supplement: Supplementary material [file KGMR_A_2607927_SM5877.zip › Fig S2.tif]

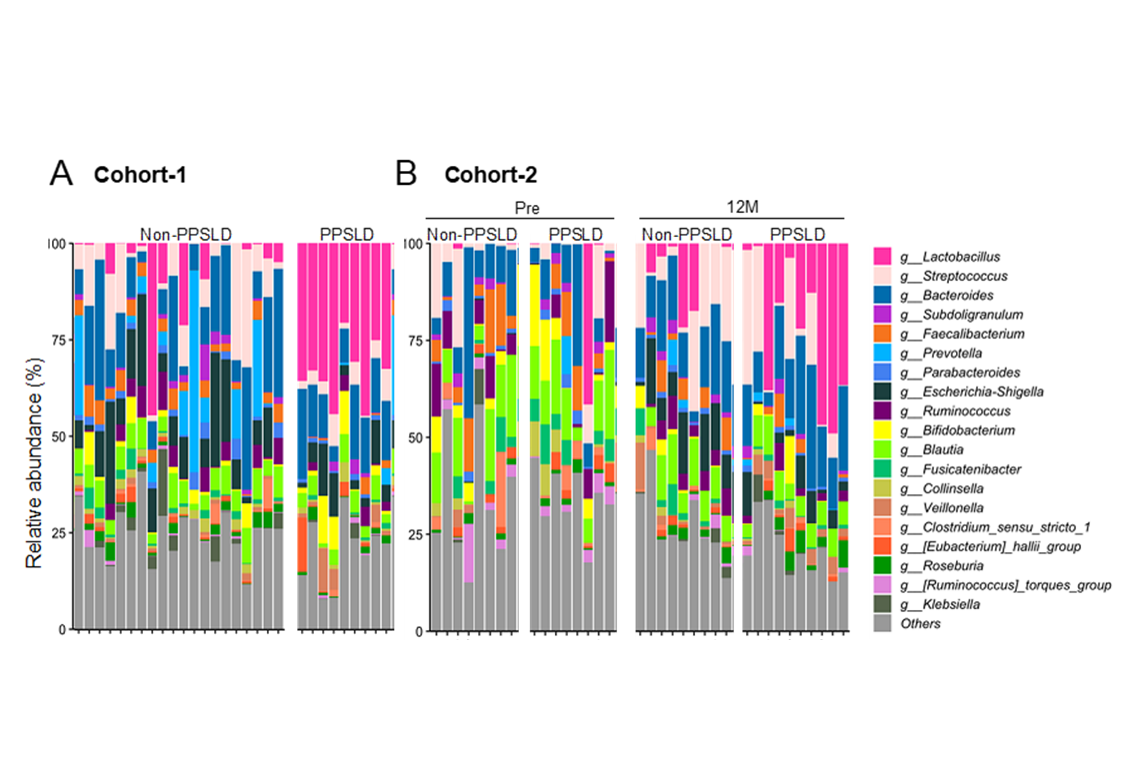

Supplement: Supplementary material [file KGMR_A_2607927_SM5877.zip › Fig S3.tif]

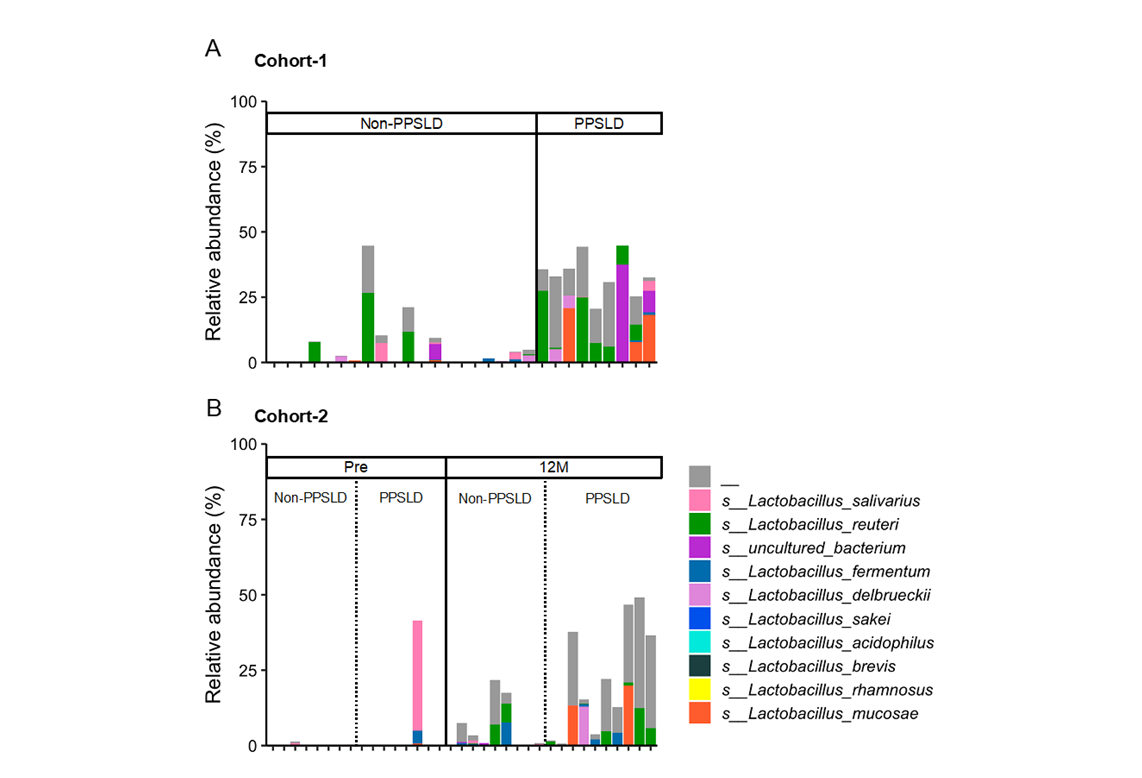

Supplement: Supplementary material [file KGMR_A_2607927_SM5877.zip › Fig S4.tif]

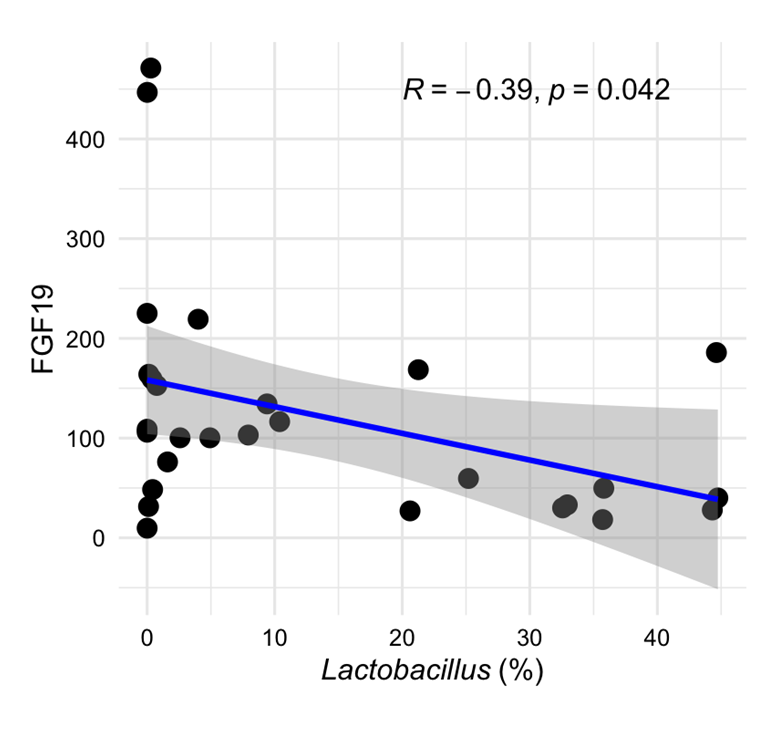

Supplement: Supplementary material [file KGMR_A_2607927_SM5877.zip › Fig S5.tif]
